# Disentangling Sum-Frequency Generation Spectra of the Water Bending Mode at Charged Aqueous Interfaces

**DOI:** 10.1021/acs.jpcb.1c03258

**Published:** 2021-06-23

**Authors:** Takakazu Seki, Chun-Chieh Yu, Kuo-Yang Chiang, Junjun Tan, Shumei Sun, Shuji Ye, Mischa Bonn, Yuki Nagata

**Affiliations:** † Max Planck Institute for Polymer Research, Ackermannweg 10, 55128 Mainz, Germany; ‡ Hefei National Laboratory for Physical Sciences at the Microscale, and Department of Chemical Physics, University of Science and Technology of China, 230026 Hefei, China; § Department of Physics and Applied Optics Beijing Area Major Laboratory, Beijing Normal University, Beijing 100875, China

## Abstract

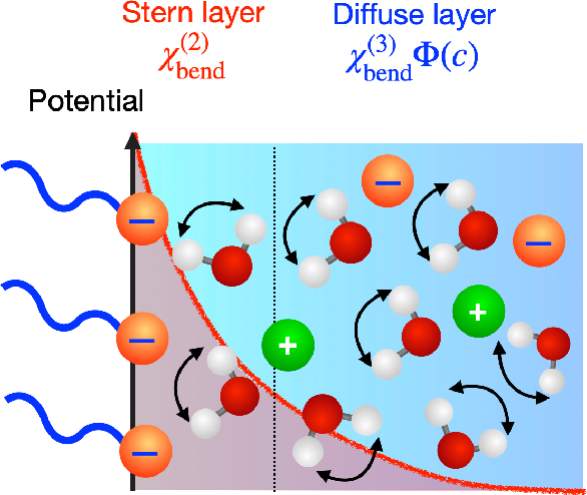

The origin of the sum-frequency generation (SFG) signal of the water bending mode has been controversially debated in the past decade. Unveiling the origin of the signal is essential, because different assignments lead to different views on the molecular structure of interfacial water. Here, we combine collinear heterodyne-detected SFG spectroscopy at the water-charged lipid interfaces with systematic variation of the salt concentration. The results show that the bending mode response is of a dipolar, rather than a quadrupolar, nature and allows us to disentangle the response of water in the Stern and the diffuse layers. While the diffuse layer response is identical for the oppositely charged surfaces, the Stern layer responses reflect interfacial hydrogen bonding. Our findings thus corroborate that the water bending mode signal is a suitable probe for the structure of interfacial water.

## I Introduction

The bending mode of H_2_O has a characteristic frequency around 1550–1700 cm^–1^. This mode has been probed using vibrational spectroscopies, because it reports on the local structure of the hydrogen-bond network in water; when water is strongly (weakly) hydrogen-bonded, the frequency of the bending mode is blue-shifted (red-shifted).^1,2^ Probing the bending mode of water has several advantages over probing the O–H stretch mode. Whereas the O–H stretch mode of water cannot be spectrally distinguished from other molecules containing OH-groups, the H–O–H water bending mode is specific to water.^3−8^ Also, the vibrational coupling between bending modes has a limited impact on its spectral response,^3,9^ in sharp contrast to the O–H stretch mode.^10,11^ Furthermore, understanding the bending mode is essential to unveil the vibrational energy transfer from the O–H stretch mode of water and the amide mode of proteins to the local heat,^12−16^ because the bending mode is believed to be an essential intermediate step to receive excess vibrational energy and release it to the local heat.^9,17−21^

The H–O–H bending mode of specifically interfacial water molecules has been probed with sum-frequency generation (SFG) spectroscopy.^3,22−28^ Although SFG spectroscopy is surface-specific, the precise origin of the SFG signal has been highly debated. So far, three distinct contributions have been proposed, from interfacial dipoles, bulk quadrupoles, and interfacial quadrupoles.^29,30^ The dipole contribution refers to the first-order term of the second-order susceptibility, and a number of research groups have analyzed and interpreted the experimental and simulated SFG data of the bending mode based on the dipole mechanism.^22−24,31−33^ The bulk quadrupole mechanism was proposed by Tahara, Morita, and co-workers in 2016, in which the first-order dipole term is masked by a higher-order term.^27^ More recently, in 2020, a new set of the bending mode SFG spectra demonstrated the frequency shift of the bending mode due to the interaction of water with lipids/surfactants. Because the frequency shift cannot be accounted for via the bulk quadrupole mechanism, Tahara and co-workers proposed that the bending mode SFG signal is generated by the higher-order term arising from the interface (interfacial quadrupole mechanism).^28^

Clarifying this apparent contradiction by unveiling the origin of the SFG signal is important, because the different assignments of the origin of the signal lead to different interpretations of the bending mode of water—and thereby of the structure of interfacial water. If the χ_bend_^(2)^ signal arises from the dipole mechanism, it provides information on the molecular orientation of the interfacial water molecules.^34^ If the signal arises through the interfacial quadrupole mechanism, one cannot obtain orientational information.^35,36^

Currently, the bulk quadrupole mechanism is not supported by any experimental data. The remaining two mechanisms, dipole mechanism and interfacial quadrupole mechanism, can be identified from the sign of the H–O–H bending mode (χ_bend_^(2)^) at the charged interfaces. If χ_bend_^(2)^ is governed by the dipole mechanism, the sign of the Im(χ_bend_^(2)^) signal changes with the sign of the surface charge. If χ_bend_^(2)^ is governed by the interfacial quadrupole mechanism, the sign of the Im(χ_bend_^(2)^) signal is positive, irrespective of the sign of the surface charge.^36^

**Figure 1:**
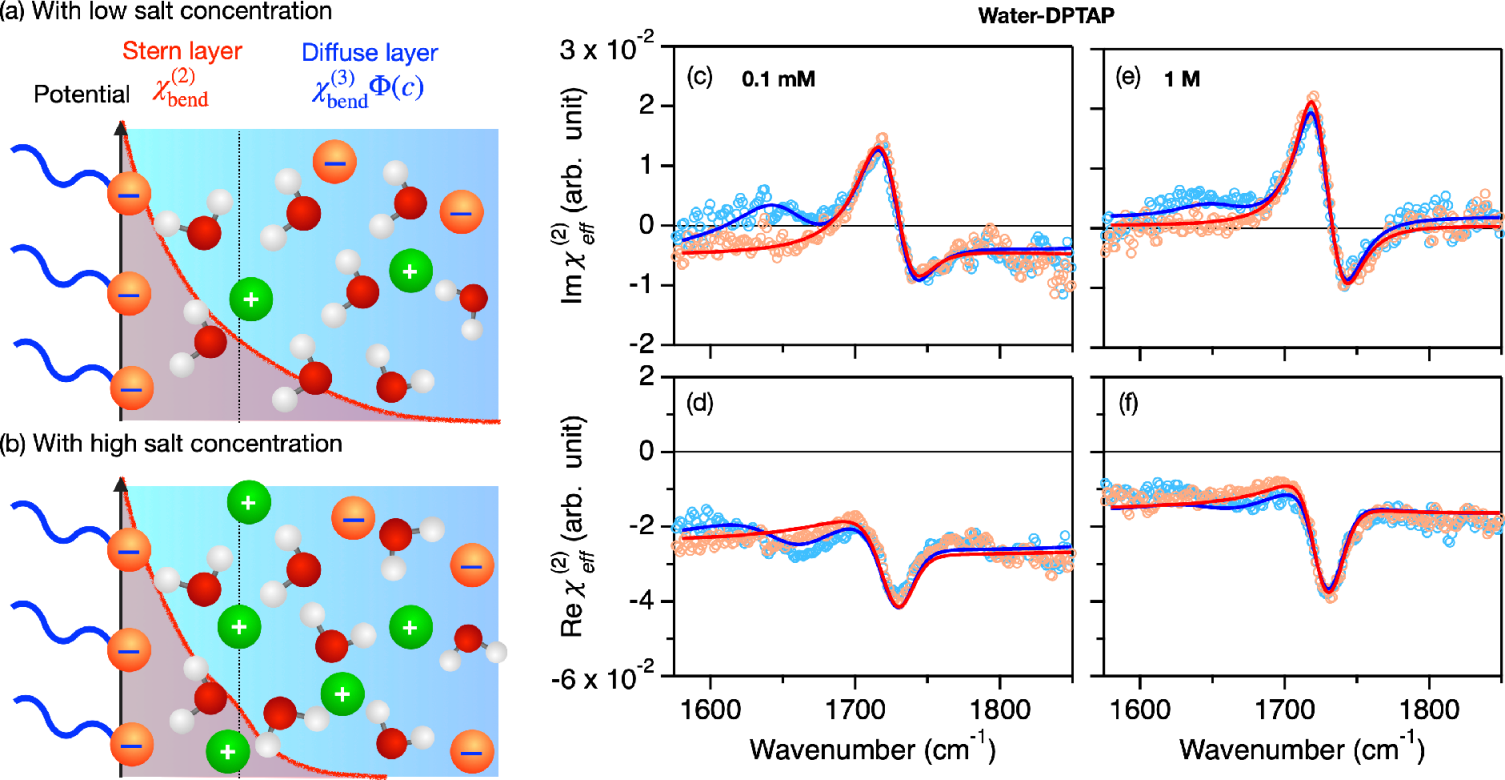
(a,b) Schematics for the Stern layer and the diffuse layer contributions corresponding the χ_bend_^(2)^ and χ_bend_^(3)^(ω)Φ(*c*) contributions, respectively, in the presence of (a) low concentration and (b) high concentration of salt. Φ(*c*) represents the surface potential as a function of salt concentration. (c–f) HD-SFG spectra at the H_2_O–DPTAP interface and at the D_2_O–DPTAP interface with two different NaCl concentrations. The blue and red data points indicate the H_2_O–DPTAP and D_2_O–DPTAP data, respectively. The solids lines represent the fits.

Extracting the χ_bend_^(2)^ contribution at the charged interfaces is, however, not straightforward because the water signal at these interfaces arises not only from the oriented water molecules in the Stern layer (χ_bend_^(2)^ term) which is invariant to the solution’s salt concentration, but also from those oriented along the interfacial electric field in the diffuse layer (χ_bend_^(3)^ term) (Figure 1a,b).^37^ This interfacial field and the magnitude of the χ_bend_^(3)^ contribution has been examined by varying the bulk electrolyte concentration.^38−40^ However, the χ_bend_^(3)^ contribution is controversial: Reference (26) indicated a substantial χ_bend_^(3)^ contribution, leading to the flipping of the sign of the Im(χ_bend_^(2)^) peak due to the change of the negatively and positively charged interfaces (dipole mechanism), while ref (28) showed that the χ_bend_^(3)^ contribution is negligible, leading to the positive Im(χ_bend_^(2)^) peak irrespective of negatively or positively charged interfaces (interfacial quadrupole mechanism).

Here, using collinear heterodyne (HD)-SFG, we measure the H–O–H bending mode of water at the positively charged lipid (1,2-dipalmitoyl-3-trimethylammonium propane, DPTAP) and negatively charged lipid (1,2-dipalmitoylsn-glycero-3-phospho-glycerol, DPPG) interfaces. We unambiguously establish that the χ_bend_^(3)^ contribution is non-negligible and determine its spectrum. The careful extraction of the Im(χ_bend_^(2)^) signal, by varying the electrolyte concentration, reveals that the sign of the Im(χ_bend_^(2)^) signal is opposite at the water–DPTAP and water–DPPG interfaces. We highlight the importance of the homogeneous sampling of the water–lipid interface, which could be achieved by rotating the sample in the collinear HD-SFG setup.

## II Methods

### II.A Sample Preparation

We dissolved DPPG (sodium salt) and DPTAP (chloride salt) purchased from Avanti Polar Lipids in a mixture of 90% chloroform (Fischer Scientific, stabilized with amylene, >99%) and 10% methanol (VWR Chemicals, 99.8%) at a concentration of 4.3 × 10^–4^ mol/L. Sodium chloride (Sigma-Aldrich, >99.5%) was baked in an oven for 8 h at 650 °C. We used D_2_O (>99.9%), which was purchased from Sigma-Aldrich. H_2_O was obtained from a Milli-Q machine (resistance >18.2 MΩ cm). We prepared the sodium chloride solutions with their concentrations of 1 M and 0.1 mM. We chose the concentrations of 0.1 mM and 1 M to see the spectral deformations in both imaginary and real parts due to the complex χ_bend_^(3)^ term, as is seen in what follows.

The 20 mL sodium chloride solutions were poured into a Teflon trough with an 8.0 cm diameter. We then deposited ∼50 μL DPTAP and DPPG solutions onto the H_2_O and D_2_O solutions using a click syringe. The surface pressure of the DPTAP and DPPG monolayers was measured with a commercial surface tension meter (Kibron, Inc., Helsinki, Finland) and was determined to be ∼44 ± 3 mN/m and 19 ± 3 mN/m, respectively. The surface area per lipid was estimated to be ∼44 Å^2^ and ∼52 Å^2^ for DPTAP and DPPG, respectively.^41,42^ The prepared samples were equilibrated for at least 40 min. For both HD-SFG and HD-SHG measurements, the trough was rotated to avoid the lipid monolayer distortion due to heat accumulation.^43^ The speed of the sample at the laser irradiation spot was ∼1.0 cm/s.

In this study, we used the charged lipids of DPPG and DPTAP with the C=O groups. The C=O stretch mode contributions interfere with the H–O–H bending mode of water,^26^ which may potentially complicate the interpretation on the SFG spectra. The other choices which have been commonly used for generating the charged surfaces are the surfactants without the C=O groups, such as sodium dodecyl sulfate (SDS) and cetyltrimethylammonium bromide (CTAB).^24,44^ However, SDS and CTAB have critical micellar concentrations of ∼0.1 mM to 1 mM, much higher than DPPG and DPTAP. In fact, for stable SFG measurements, researchers have used 1 mM–10 mM concentrations of SDS and CTAB.^44−46^ Such high SDS and CTAB bulk concentrations prohibit fine control of charge screening by sodium chloride to tune the χ_bend_^(3)^ contribution, which requires concentrations down to 0.1 mM. The bulk concentrations of the DPPG and DPTAP samples were ∼1 μM, much smaller than the 0.1 mM salt concentration. For DPPG and DPTAP, one can control the ionic strength with the salt concentration, allowing us to uncover the χ_bend_^(3)^ contribution, unlike SDS and CTAB.

### II.B HD-SFG Measurements

The HD-SFG measurements were performed on a collinear beam geometry using a Ti:Sapphire regenerative amplifier (Spitfire Ace, Spectra-Physics, centered at 800 nm, ∼40 fs pulse duration, 5 mJ pulse energy, 1 kHz repetition rate). The visible and IR beams were first focused into a 20 μm-thick y-cut quartz plate to produce sum-frequency signal serving as local oscillator (LO). These beams were then collinearly passed through an 8 mm CaF_2_ plate for the phase modulation and focused on the sample surface at an angle of 45°. The SFG signal from the sample interferes with the SFG signal from the LO, generating the SFG interferogram. The SFG interferogram was dispersed in a spectrometer and detected by an EMCCD camera. The complex χ_eff_^(2)^ spectra were obtained via the Fourier analysis of the interferogram and normalization by a *z*-cut quartz crystal. The measurements were performed with *ssp* (denoting *s*-, *s*-, and *p*-polarized SFG, visible, and IR beams, respectively) polarization combination. The details of the HD-SFG setup can be found in the Supporting Information.

Note that the HD-SFG measurement for the rotating sample is challenging because the height of the sample fluctuates due to the rotation of the sample, causing phase modulations. The height fluctuation of our samples had a standard deviation of 1.7 μm, which will cause ∼6° phase error with a typical noncollinear SFG setup.^47^ In this work, we used a collinear HD-SFG geometry, which is much less sensitive to the height change than the noncollinear HD-SFG setup, and thus the phase error for the rotating sample is <1.7°.^48−50^ Such a collinear HD-SFG setup is thus very suitable for HD-SFG measurements of rotating samples.

### II.C HD-SHG Measurements

A pulsed Yb:KGW (ytterbium-doped potassium gadolinium tungstate) laser system (Pharos, Light Conversion Ltd.) was used, generating pulses with a wavelength of ∼1030 nm, a pulse duration of roughly 210 fs, a repetition rate of 1 MHz, and a pulse energy of 15 μJ. The pulse energy was reduced to 300 nJ. After passing through *y*-cut quartz to generate the LO signal, and fused silica plates for phase modulation, the fundamental beam was focused onto the sample surface. All the measurements were performed with *s*-in/*p*-out polarization combinations. The incident angle of the incoming beam was set to 45° relative to the surface normal. The generated second harmonic generation (SHG) signal was dispersed in a spectrograph and detected by an EMCCD camera.

## III Results and Discussion

### III.A Evidence of Non-negligible χ_bend_^(3)^ Contribution

Figures 1c,f display the complex SFG susceptibility (χ_eff_^(2)^) at the D_2_O–DPTAP and H_2_O–DPTAP interfaces with two different salt concentrations. First, we focus on the Im(χ_eff_^(2)^) spectra at the D_2_O–DPTAP interface. For D_2_O, the bending mode is shifted to ∼1200 cm^–1^, outside the studied frequency window, so that these measurements serve as a reference. For all the salt concentrations, the spectra commonly show a large positive peak at ∼1720 cm^–1^ and a relatively small negative peak at ∼1740 cm^–1^. These peaks are attributed to the C=O stretch mode.^51^ A striking change of the spectra with increasing salt concentration is the elevation of the baseline (frequency-independent nonresonant contribution). We then turn our focus to the Im(χ_eff_^(2)^) spectra of the H_2_O–DPTAP samples. Here, the H–O–H bending mode appears as a 1650 cm^–1^ peak feature.^26,28^ Upon increasing the salt concentration from 0.1 mM to 1 M, the 1650 cm^–1^ peak varies substantially.

**Figure 2:**
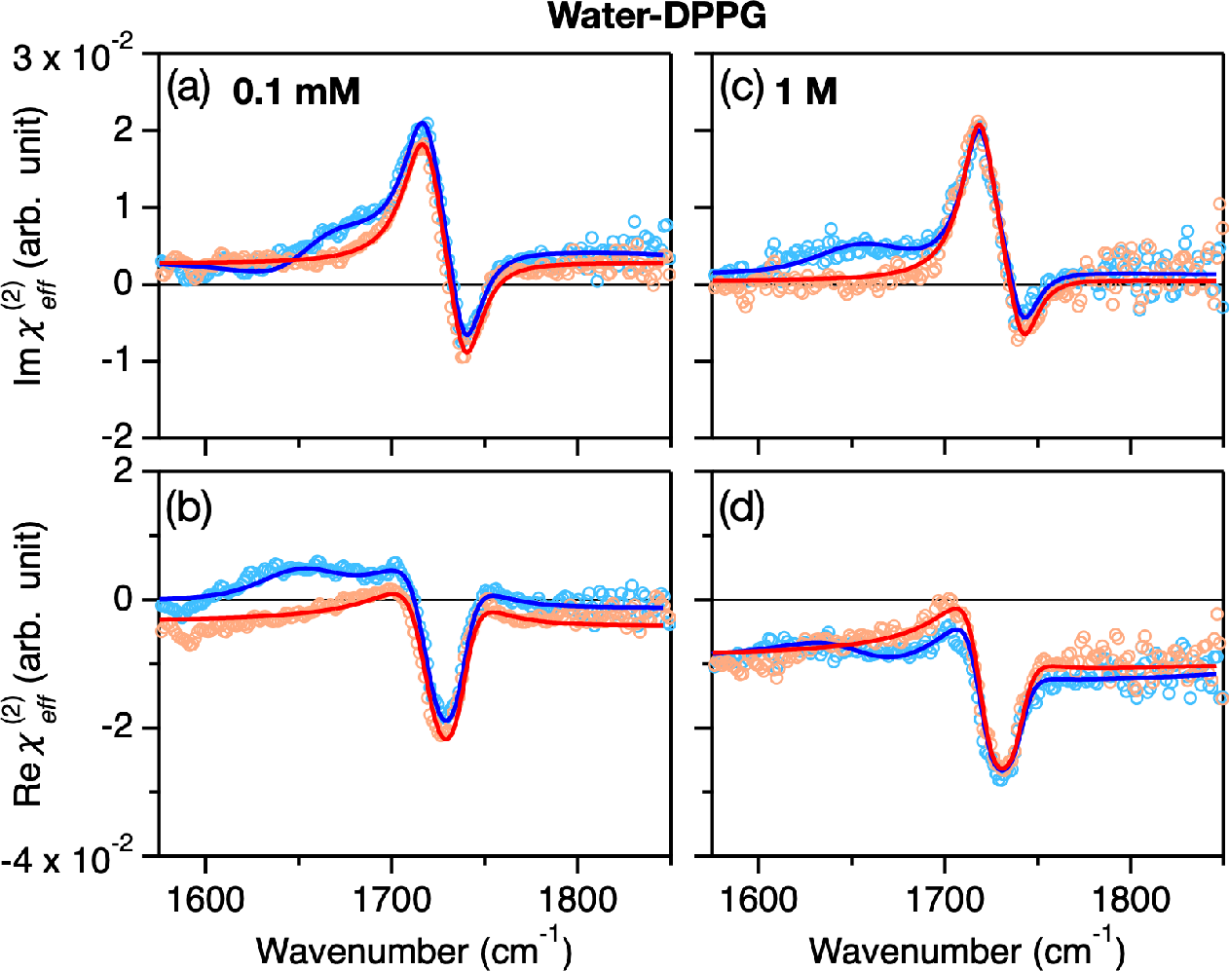
(a–d) HD-SFG spectra at the H_2_O–DPPG and D_2_O–DPPG interfaces with two different NaCl concentrations. The blue and red data points indicate H_2_O–DPPG and D_2_O–DPPG data, respectively. The solids lines represent the fits.

Subsequently, we measured the χ_eff_^(2)^ spectra of the H_2_O- and D_2_O-negatively charged DPPG samples. The spectra are shown in Figure 2. The D_2_O–DPPG samples also show the positive 1720 cm^–1^ and negative 1740 cm^–1^ C=O stretch features, while the H_2_O–DPPG samples possess the ∼1650 cm^–1^ H–O–H bending mode contribution, in addition to the C=O stretch features. Again, upon changing the salt concentration, the 1650 cm^–1^ peak varies.

The SFG response of the H_2_O and D_2_O samples in the measured frequency range can be approximated by
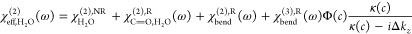
1
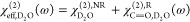
2respectively, where χ^(2),NR^ represents the nonresonant contribution, χ_C=O_^(2),R^ (ω) denotes the resonant contribution from the C=O stretch mode. *c*, Φ, κ, and Δ*k*_*z*_ denote ion concentration, the surface potential, the inverse of the Debye length, and the mismatch of the wave-vectors along the surface normal in the reflected SFG configuration, respectively.^26,38^ By assuming that χ_H_2_ O_^(2),NR^(ω) = χ_D_2_O_^(2),NR^(ω) and χ_C=O,H_2_ O_^(2),R^(ω) = χ_C=O,D_2_O_^(2),R^(ω), that is, the nuclear quantum effects (NQEs) are negligible, one can get the H–O–H bending mode contribution 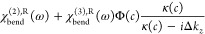
by subtracting χ_eff,D_2_O_^(2)^(ω) from χ_eff,H_2_O_^(2)^(ω). Here, we assumed negligible NQEs on the spectral shape, in analogy with previous work.^28^ We will discuss the validity of this assumption in the following.

**Figure 3:**
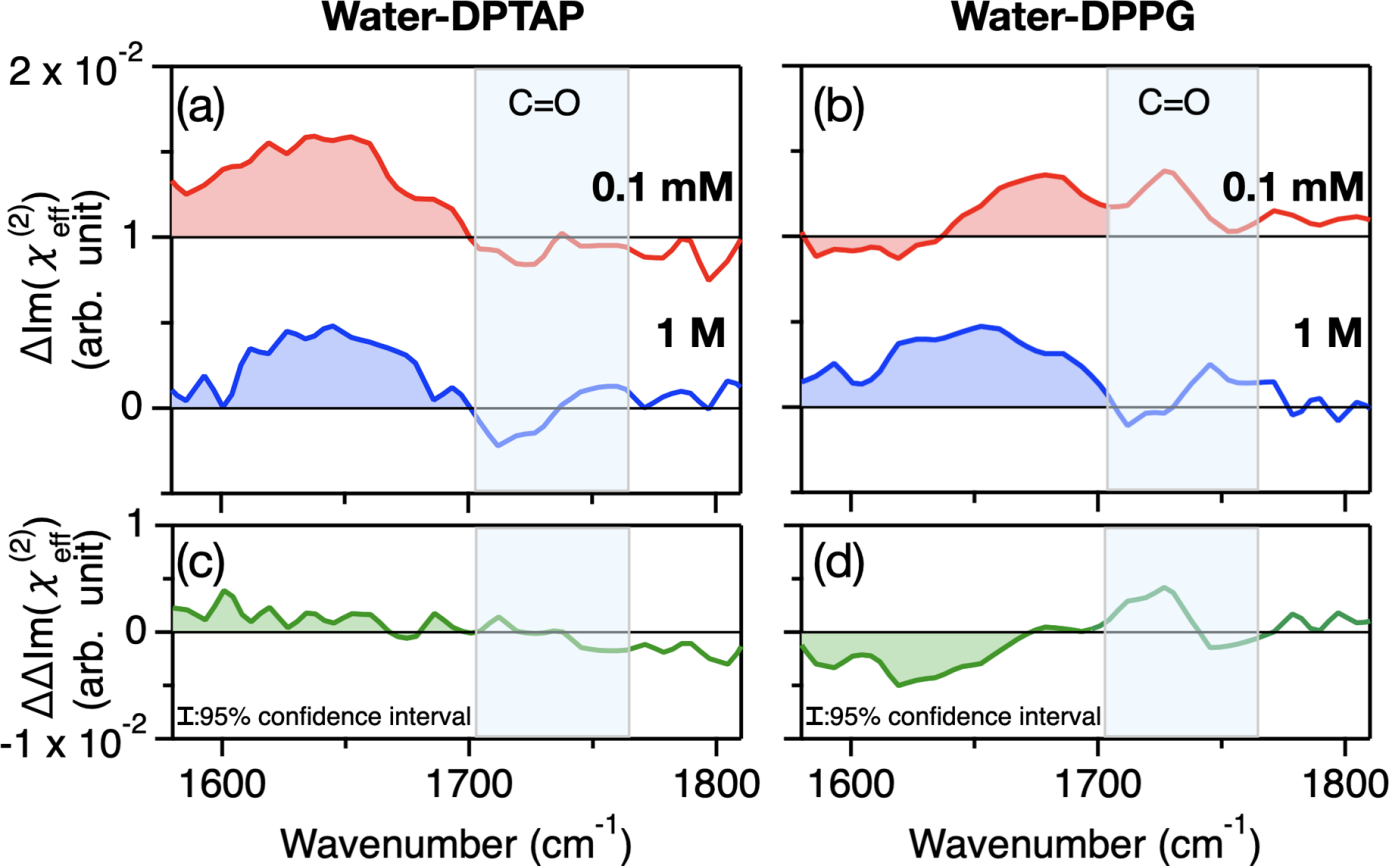
(a,b) The ΔIm(χ_eff_^(2)^(ω,*c*)) spectra obtained through the subtraction of D_2_O data from the H_2_O data of the (a) DPTAP and (b) DPPG samples at two different salt concentrations. The spectra are offset by 0.01 for clarity. (c,d) The ΔΔIm(χ_eff_^(2)^(ω)) spectra for the (c) DPTAP and (d) DPPG interfaces obtained through the subtraction of ΔIm(χ_eff_^(2)^(ω,*c* = 1M) spectrum from ΔIm(χ_eff_^(2)^(ω,*c* = 0.1 M) spectrum. The features appearing in the region shaded in light blue result from the residual C=O contributions. The dotted lines indicate the residual nonresonant contribution inferred from the fits.

Figure 3 panels a and b show the subtracted spectra (ΔIm(χ_eff_^(2)^(ω,*c*)) = Im (χ_eff,H_2_ O_^(2)^(ω,*c*)) – Im (χ_eff,D_2_O_^(2)^(ω,*c*))) of the DPTAP and DPPG samples, respectively. The ΔIm(χ_eff_^(2)^(ω,*c*)) response in the 1580–1630 cm^–1^ frequency region decreases for the DPTAP sample, when the salt concentration increases from *c* = 0.1 mM to 1 M. On the other hand, the ΔIm(χ_eff_^(2)^(ω,*c*)) response in the 1580–1630 cm^–1^ frequency region increases for the DPPG sample. The changes of the ΔIm(χ_eff_^(2)^ (ω,*c*)) spectra with varying salt concentration signify the non-negligible ΔIm(χ_bend_^(3),R^(ω) contribution.

We further calculated the spectra ΔΔIm(χ_eff_^(2)^(ω)) = ΔIm(χ_eff_^(2)^(ω,*c* = 0.1 mM)) – ΔIm(χ_eff_^(2)^(ω,*c* = 1 M)), which reflect the  contribution (again under the assumption of negligible NQEs). The data are shown in Figure 3c,d for the DPTAP and DPPG samples, respectively. The ΔΔIm(χ_eff_^(2)^(ω)) spectra showed a positive 1580–1670 cm^–1^ contribution for the DPTAP sample and a negative contribution for the DPPG sample. The ΔΔIm(χ_eff_^(2)^(ω)) contribution in the ω < 1650 cm^–1^ region is more apparent than that in the ω > 1650 cm^–1^ region, where 1650 cm^–1^ is a typical H–O–H bending mode frequency. The prominent ΔΔIm(χ_eff_^(2)^(ω)) contribution in the ω < 1650 cm^–1^ region arises from the  term.

The positive and negative ΔΔIm(χ_eff_^(2)^(ω)) contributions for the DPTAP and DPPG samples indicate that the ΔΔIm(χ_eff_^(2)^(ω)) signal is governed by the 
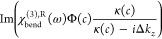
term. Since the sign of the 
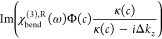
varies with the sign of the surface charge due to the surface potential of Φ(*c*), the flipping of the sign for ΔΔIm(χ_eff_^(2)^(ω)) for the positively charged DPTAP and negatively charged DPPG surface provides direct evidence for the χ_bend_^(3),R^(ω) contribution.

The current finding is at odds with that in ref (28) in which the H–O–H bending mode contribution is unchanged upon the addition of the salt. The reason for such a discrepancy may be attributable to the lipid monolayer formation. A lipid monolayer is easily displaced from the laser spot as a result of the heat accumulation due to continued laser irradiation.^43^ Because we used the rotating trough, such heat accumulation can be avoided.

This hypothesis can be confirmed by investigating the SFG signature of the C=O stretch mode at the H_2_O–DPTAP and D_2_O–DPTAP interfaces. First, the ratio of the C=O stretch peak amplitude vs the H–O–H bending mode amplitude in the Im(χ_eff_^(2)^) spectrum at the H_2_O–DPTAP interface is much larger in this work than that reported in ref (28). This implies that the coverage of the DPTAP is higher in this work than in ref (28). Furthermore, the C=O peak frequency is ∼1730 cm^–1^ in the intensity |χ_eff_^(2)^|^2^ spectra at the D_2_O–DPTAP interface in refs (51 and 26), as well as our measurement (see Supporting Information), while the C=O peak is located at ∼1740 cm^–1^ in ref (28). Because the lower surface coverage of DPTAP results in the blue-shift of the C=O stretch peak,^51^ the 1740 cm^–1^ C=O peak observed in ref (28) indicates that the coverage of the DPTAP is likely strongly reduced in the probed region. With decreasing surface coverage of DPTAP, the surface charge decreases, lowering the impact of the χ_bend_^(3)^ contribution on the SFG spectra. Note that very recently, Bakker and co-workers also pointed out that too small a surface charge leads to negligibly small dipolar contribution of the bending mode in ref (44).

### III.B Determination of χ_bend_^(2)^ and χ_bend_^(3)^ Spectra

**Figure 4:**
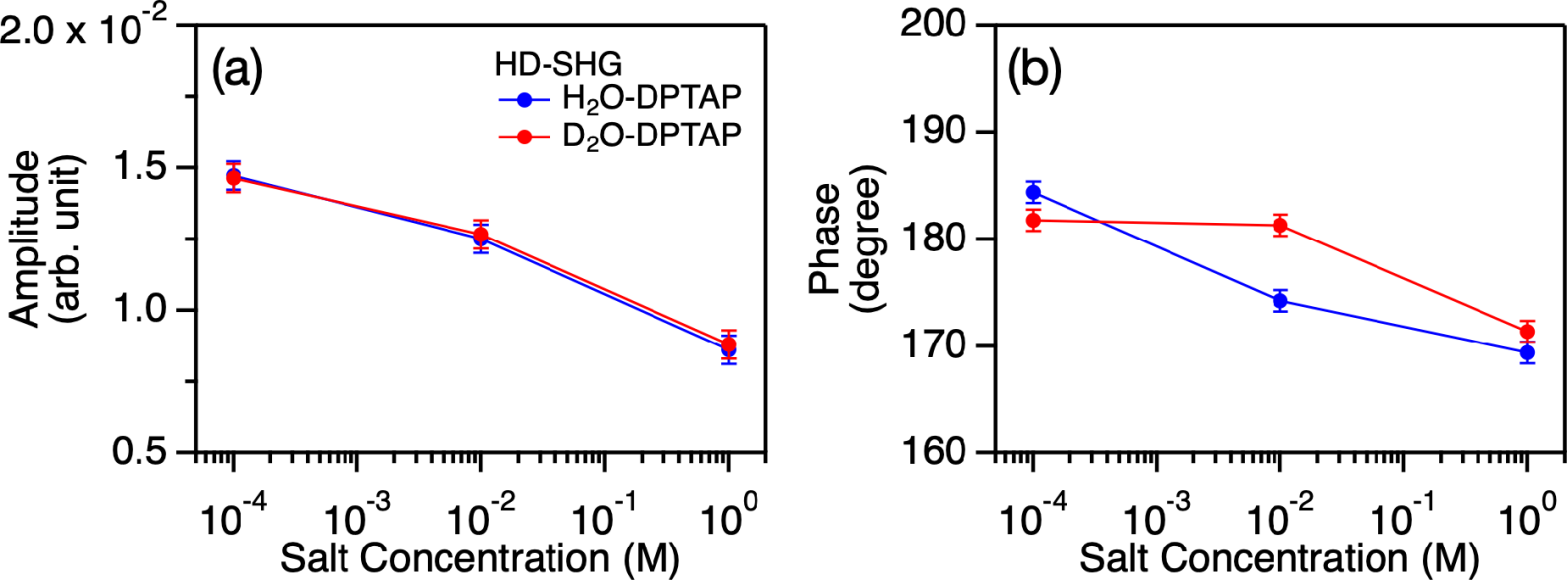
(a,b) Amplitude (a) and phase (b) of the nonresonant contribution obtained by HD-SHG for water–DPTAP interface with various salt concentrations. The error bars indicate the 95% confidence intervals. The nonresonant contribution estimated from the fits of the HD-SFG data was also compared in the Supporting Information.

The above result of the significant χ_bend_^(3)^ contribution manifests that the χ_bend_^(2)^ and χ_bend_^(3)^ contributions are entangled in the measured χ_eff_^(2)^ spectra. Thus, disentangling the χ_bend_^(2)^ contribution from the χ_bend_^(3)^ contribution requires fitting of the spectra. Here, before carrying out the fitting, we verify the assumption that the NQE is negligible between the H_2_O and D_2_O samples. In fact, the different nonresonant background between the H_2_O and D_2_O samples can be seen in the nonzero ∼1800 cm^–1^ region of ΔIm(χ_eff_^(2)^(ω,*c*)) of the DPTAP and DPPG samples as well as the SDS data in ref (28). Because the nonresonant contribution critically affects the inferred amplitude of the H–O–H bending mode signal, we checked whether the NQEs differentiate the nonresonant background of the H_2_O and D_2_O samples at the water–DPTAP interface by using HD-SHG spectroscopy. The amplitudes and phases obtained in the HD-SHG measurements are plotted in Figure 4 panels a and b, respectively, for DPTAP. The amplitude of the nonresonant contribution is very similar for the H_2_O and D_2_O samples, while the phase differs significantly, particularly for the 10 mM salt concentration. Currently, we are not sure how the NQEs affect the nonresonant contributions. Simulation techniques, including the NQEs,^52^ may be able to clarify the origin of the difference between the H_2_O and D_2_O samples.

On the basis of the knowledge that the nonresonant contributions of the H_2_O sample χ_H_2_O_^(2),NR^) and the D_2_O sample (χ_D_2_O_^(2),NR^) can be different, we extracted the χ_bend_^(2)^(ω) and χ_bend_^(3)^(ω) contributions from the SFG spectra at the DPTAP and DPPG interfaces. For fitting the H–O–H bending mode contribution, we used a Voigt profile;^53,54^
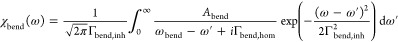
3where *A*_bend_, ω_bend_, and Γ_bend,hom_ denote, respectively, the amplitude, characteristic frequency, and line width associated with homogeneous broadening, and Γ_bend,inh_ accounts for inhomogeneous broadening. For the fit of the C=O stretch modes, we used the two Lorentzian functions corresponding to the positive and negative features.^51^

We performed the global fitting for all 16 spectra (H_2_O/D_2_O × two different salt concentration × imaginary/real parts × DPTAP/DPPG). Here, χ_bend_^(3)^(ω) was kept fixed across all eight H_2_O spectra, because χ_bend_^(3)^(ω) reflects the bulk water properties and should thus be independent of the lipid species. The four H_2_O–DPTAP spectra and the four H_2_O–DPPG spectra each had one fixed χ_bend_^(2)^(ω), as the Stern layer contribution is largely independent of the ionic strength and thus is insensitive to the salt concentration. Furthermore, the parameters for the C=O stretch modes were identical between H_2_O and D_2_O samples. The global fitting provides the robust estimation of the χ_bend_^(2)^(ω) and χ_bend_^(3)^(ω) contributions. The details of the fitting functions and obtained parameters can be found in the Supporting Information.

**Figure 5:**
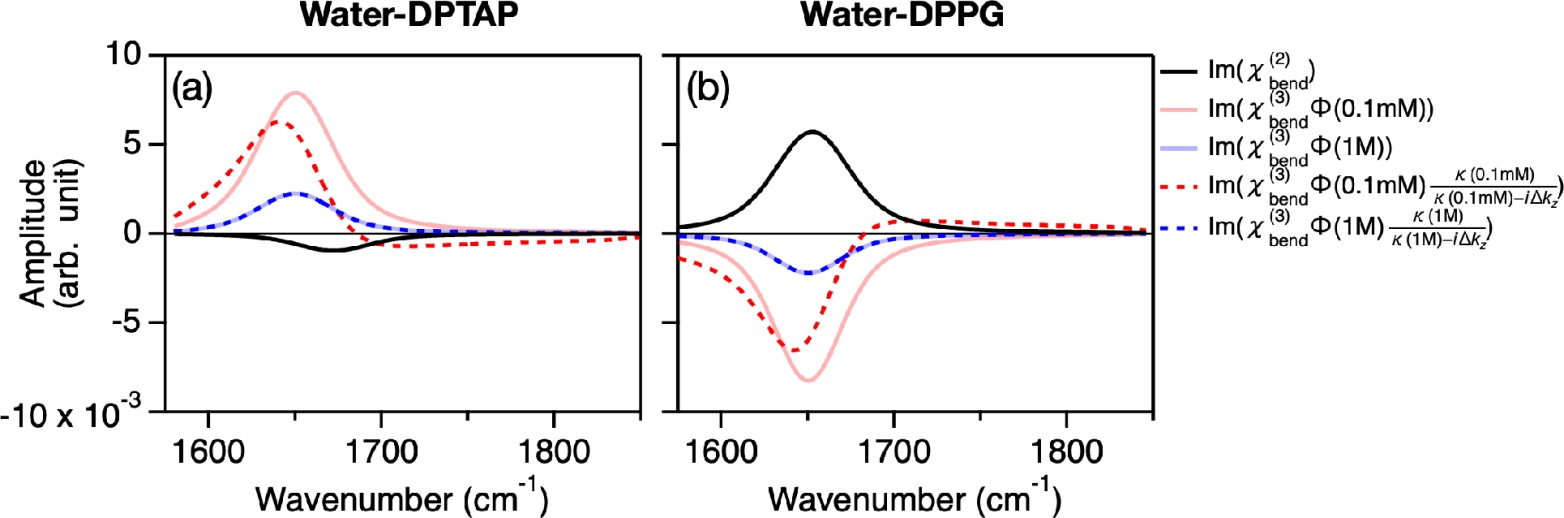
(a,b) Im(χ_bend_^(2)^(ω)), Im(χ_bend_^(3)^(ω)Φ(*c*)), and Im(χ_bend_^(3)^(ω)Φ(*c*)(κ(*c*)/(κ(*c*) – *i*Δ*k_z_*))) spectra obtained from the fit for (a) water-DPTAP and (b) water-DPPG interfaces, respectively. The Im(χ_bend_^(2)^(ω)) and Im(χ_bend_^(3)^(ω)Φ(*c*))/Im(χ_bend_^(3)^(ω)Φ(*c*)(κ(*c*)/(κ(*c*) – *i*Δ*k_z_*))) contributions indicate the Stern layer contribution and the diffuse layer contribution, respectively. The Im(χ_bend_^(3)^(ω)Φ(*c*)(κ(*c*)/(κ(*c*) – *i*Δ*k_z_*))) contribution includes the phase mismatching term, showing the effective diffuse layer contribution in the Im(χ_eff,H_2_O_^(2)^(ω)) spectra.

The obtained fits are plotted in the solid lines of Figures 1(c–f) and 2, while the χ_bend_^(2)^(ω) and χ_bend_^(3)^(ω)Φ(*c*) spectra obtained from the fit are shown in Figure 5. The inferred Im(χ_bend_^(2)^(ω)) and Im(χ_bend_^(3)^(ω)Φ(*c*)) spectra are negative and positive for the DPTAP samples, while these are positive and negative for the DPPG samples. The mechanism of the opposite sign of the χ_bend_^(2)^(ω) and χ_bend_^(3)^(ω)Φ(*c*) contributions was previously explained using *ab initio* calculations.^26^ The  term causes a line shape modulation of Im(χ_bend_^(3)^(ω)Φ(*c*)) spectra in the low concentration regime (red dotted lines in Figure 5), giving rise to the low frequency contribution in the frequency region of less than ∼1650 cm^–1^, as discussed above. We would like to stress that the opposite signs of the Im(χ_bend_^(2)^(ω)) peak at the positively charged DPTAP and the negatively charged DPPG interfaces reveal that the H–O–H bending mode SFG feature arises from the dipole rather than from the quadrupole contribution.

The peak frequencies of the χ_bend_^(2)^(ω) contribution at the H_2_O–DPTAP and H_2_O–DPPG interfaces were 1672 ± 10 and 1652 ± 2 cm^–1^, respectively. Since a higher bending mode frequency indicates a stronger hydrogen bond,^1^ the peak frequencies indicate that a water molecule in the vicinity of the DPTAP interface has stronger hydrogen bonding than those at the DPPG interface. This trend is consistent with the O–H stretch data of the DPTAP and DPPG interface; the HD-SFG spectra show that the H_2_O–DPTAP interface (center-of-mass frequency of 3360 cm^–1^) shows a slightly lower frequency than the H_2_O–DPPG interface (3390 cm^–1^).^55^ The slightly higher frequency of the H_2_O molecules near the PO_4_^–^ part of the phospholipid can be rationalized by previous simulation data.^56^ This qualitative agreement between interfacial water stretch and bend frequencies substantiates the conclusion that the χ_bend_^(2)^(ω) response originates from the interfacial dipole. As such, the χ_bend_^(2)^(ω) peak contains information on the hydrogen bond structure of the interfacial water molecules.

The χ_bend_^(2)^(ω) contribution has a peak frequency of 1650 cm^–1^ and a full-width at half-maximum (fwhm) of ∼60 cm^–1^. Since the χ_bend_^(3)^(ω) contribution reflects the bulk properties, the peak frequency and fwhm of the χ_bend_^(3)^(ω) spectra can be compared with the IR and Raman spectra of the water bending mode. Indeed, these values are very comparable to the 1644 cm^–1^ peak frequency and ∼70 cm^–1^ fwhm of the IR spectrum of the water bending.^9^

## IV Conclusions

We performed the HD-SFG measurement of the H–O–H bending mode of water at the water-positively charged DPTAP and water-negatively charged DPPG interfaces. Our data show that the χ_bend_^(3)^(ω) contributions are not negligible at the charged interface. The sign of the Im(χ_bend_^(2)^(ω)) spectrum at the water–DPTAP interface is negative, whereas the sign of the Im(χ_bend_^(2)^(ω)) spectrum at the water–DPPG interface is positive. The change of the peak sign indicates that the bending mode signal arises from the dipole mechanism. Furthermore, we discussed the obtained frequency for the Im(χ_bend_^(2)^(ω)). The sensitivity of the peak frequency at the different interfaces indicates that the bending mode of the interfacial water molecules can be a reporter for the hydrogen bonding structure at the interfaces.

## Supporting Information Available

Further details for the experimental methods; discussion on frequency variations of the C=O stretch mode between different research groups; fitting procedures; comparison of HD-SHG and HD-SFG data; phase-accuracy of HD-SFG measurements (PDF)
