# Supplementary material for: Disentangling Sum-Frequency Generation Spectra of the Water Bending Mode at Charged Aqueous Interfaces
Source: J Phys Chem B. 2021 Jun 23;125(25):7060–7. doi: 10.1021/acs.jpcb.1c03258 (PMC8279539; doi:10.1021/acs.jpcb.1c03258)
Supplement: Supplementary file 1 — jp1c03258_si_001.pdf [file jp1c03258_si_001.pdf]

Supporting Information: Disentangling Sum-Frequency Generation Spectra of the Water Bending Mode at Charged Aqueous Interfaces

Takakazu Seki, [1,§] *Chun-Chieh Yu,* [1,§] *Kuo-Yang Chiang,*[1] *Junjun Tan,*[2] *Shumei Sun,*[3] *Shuji Ye,*[2] *Mischa Bonn,*[1] *and Yuki Nagata*[1,*]

1. Max Planck Institute for Polymer Research, Ackermannweg 10, 55128 Mainz, Germany
2. Hefei National Laboratory for Physical Sciences at the Microscale, and Department of Chemical Physics, University of Science and Technology of China, 230026 Hefei, China.
3. Department of Physics and Applied Optics Beijing Area Major Laboratory, Beijing Normal University, Beijing 100875, China

* Correspondence: nagata@mpip-mainz.mpg.de

§: These authors contributed equally.

HD-SFG Measurements (Nagata/Bonn group)

The HD-SFG measurements were performed on a collinear beam geometry using a Ti: Sapphire regenerative amplifier (Spitfire Ace, Spectra-Physics, centered at 800 nm, ~40 fs pulse duration, 5 mJ pulse energy, 1 kHz repetition rate). A part of the output was used to generate a broadband infrared (IR) pulse in an optical parametric amplifier (Light Conversion TOPAS-C) with a silver gallium disulfide ($AgGaS_2$) crystal. The other part of the output was directed through a pulse shaper consisting of a grating-cylindrical mirror system to generate a narrowband visible pulse with a bandwidth of ~10 cm^{-1}. The IR and

visible beam were firstly focused into a 20 μm-thick y-cut quartz plate as the local oscillator (LO). Then these beams were collinearly passed through an 8 mm CaF_2 plate for the phase modulation and focused onto the sample surface at angles of incidence of 45°. The SFG signal from the sample is interfered with the SFG signal from the LO, generating the SFG interferogram. The SFG interferogram was dispersed in a spectrometer (Andor Technology, Shamrock 193i) and detected by an EMCCD camera (Andor Technology, Newton). The complex spectra of second-order nonlinear susceptibility ($\chi_{\mathrm{eff}}^{(2)}$) were obtained via the Fourier analysis of the interferogram and normalization by a z-cut quartz crystal. The measurements were performed with *ssp* (denoting *s*-, *s*-, and *p*-polarized SFG, visible, and IR beams, respectively) polarization combination.

<u>HD-SHG Measurements (Nagata/Bonn group)</u>

In the HD-SHG experiments, a pulsed Yb:KGW (ytterbium-doped potassium gadolinium tungstate) laser system (Pharos, Light Conversion Ltd) was used, generating pulses with a wavelength of ~1030 nm, a pulse duration of roughly 210 fs, a repetition rate of 1 MHz and a pulse energy of 15 μJ. The pulse energy was set to 300 nJ. The fundamental beam, after passing through a *y*-cut quartz acting as a LO and fused silica plates for phase modulation, was focused onto the sample surface. All the measurements were performed with *s*-in/*p*-out polarization combinations. The incident angle of the incoming beam was set to 45° relative to the surface normal. The generated SHG beam was dispersed in a spectrograph (Princeton Instruments, Acton SP2300) and detected by a EMCCD (Andor Technology, iXon). The complex spectra of second-order nonlinear susceptibility ($\chi_{\mathrm{eff}}^{(2)}$) were obtained via the operation of Fourier transformation, filtering the time-domain signal, and inverse Fourier transformation. The obtained spectra of the sample were normalization by the spectra for the *z*-cut quartz crystal.

Sample Preparation (Nagata/Bonn group)

For the lipid solution, we dissolved DPPG (sodium salt) and DPTAP (chloride salt) purchased from Avanti Polar Lipids in a mixture of 90% chloroform (Fischer Scientific, stabilized with amylene, >99%) and 10% methanol (VWR Chemicals, 99.8%) at a concentration of 4.3×10^{-4} mol/L. Sodium chloride (Sigma Aldrich, >99.5%) was baked in an oven for 8 hours at 650 °C. We used D_2O (>99.9%), which was purchased from Sigma-Aldrich. We used H_2O, which was obtained from a MilliQ machine (the resistance of 18.2 MΩ cm). We dissolved salt into the H_2O and D_2O and made the salt solutions.

The 20 mL salt solutions were poured into a Teflon trough with a diameter of ~8.0 cm. Subsequently, we added the lipid solution onto the H_2O and D_2O solutions using a click syringe. The surface pressure of the DPTAP and DPPG monolayers were measured with a commercial surface tension meter (KBN 315 Sensor Head, Kibron, Inc., Helsinki, Finland) and were determined to be ~44 ± 3 mN/m and ~19 ± 3 mN/m, respectively. The surface area per lipid was estimated to be 44 $Å^2$ and 52 $Å^2$ for DPTAP and DPPG, respectively[1,2]. The prepared samples were equilibrated over at least 40 min. For both HD-SFG and HD-SHG measurements, the trough was rotated with a speed of ~1.0 cm per sec with respect to the laser irradiation spot (Fig. S1). The rotating trough was needed to avoid the heat accumulation, which may cause the Marangoni flow and thus alter the lipid conformation.[3]

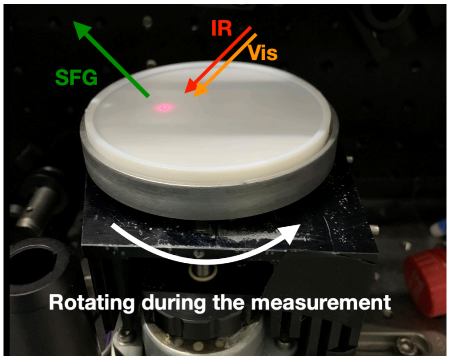

Figure S1. Photograph of the rotating trough and beam position on the sample.

We measured the fluctuation of the height due to the rotating stage via the Keyence height displacement sensor (LK-G82). The standard deviation of the height was 1.7 μm. Since the non-collinear HD-SFG measurement requires the accuracy of 1 μm,[4] the combination of the rotating trough with the collinear HD-SFG setup provides the unique solution to measure the water-lipid interface by avoiding heat accumulation.

Homodyne-SFG Measurements with Picosecond Laser Setup (Ye group)

The homodyne-SFG measurements were carried out using a commercial EKSPLA SFG system with a repetition rate of 10 Hz and a pulse width of 30 picoseconds. The SFG spectra were collected by overlapping a visible and a tunable IR beam on the sample surface using a co-propagating configuration. The visible beam (ω_{Vis}) was set at a fixed wavelength of 532.1 nm while the IR beam (ω_{IR}) can be tuned from 1500 to 1800 cm^{-1}. The angles of incidence are 63° and 53° for the visible and IR laser beams, respectively. The *ssp* SFG spectra from the water-lipid interface were collected with the spectral resolution of 5 cm^{-1}. Each spectral data point in the SFG spectra was averaged 100 shots and normalized by the intensities of the input IR and visible beams before collecting the next data point. The experiments were carried out at room temperature (25 ^{o}C).

Sample Preparation (Ye group)

Lipid DPTAP was purchased from Avanti Polar Lipids (Alabaster, AL). Sodium chloride (>99.8%) and D_2O (>99.9%) were purchased from Aldrich. DPTAP was dissolved in chloroform (purchased from Sinopharm Chemical Reagent Co., Ltd.) at a concentration of 1.0 mg/mL. The salt was baked at around 500 °C for more than 6 hours to remove the organic impurities. The ultrapure water (the resistance of 18.2 MΩ cm) was produced by Millipore system (Millipore, Bedford, MA).

The ~25 mL salt solutions were poured into a Teflon trough with a diameter of ~8.0 cm. Subsequently, we added the DPTAP lipid solution onto the H_2O and D_2O solutions

using a click syringe to get a target surface pressure of ~44 mN/m, which was measured with a commercial surface tension meter (BZY-A, Shanghai Fangrui Instrument, Inc., Shanghai, China). It was then given ~30 minutes to allow solvent evaporation and to reach surface pressure equilibrium. The trough was rotated with a speed of ~18 degree/sec to avoid heat accumulation due to the laser irradiation spot (similar to Fig. S1).

Frequency variation of the C=O Stretch Mode

In Figure S2, we compare the characteristic frequency of the C=O stretch mode in the $\left|\chi_{\mathrm{eff}}^{(2)}\right|^2$ spectra measured by the Nagata/Bonn group (Current work and Ref. 5) and that reported in Ref. 6 for the D_2O-DPTAP interface. The constructed $\left|\chi_{\mathrm{eff}}^{(2)}\right|^2$ data (red line in Fig. S2) from the HD-SFG measurement is consistent with the homodyne-detected $\left|\chi_{\mathrm{eff}}^{(2)}\right|^2$ data (orange line in Fig. S2), though the geometries of the setups and the lipid density are slightly different.[7] The C=O stretch mode frequency is ~1730 cm^{-1} for both data. On the other hand, these data differ from data reported by the Tahara group (green line in Fig. S2). The C=O stretch mode frequency is ~1740 cm^{-1} for the data obtained by Tahara group. Because the lower surface coverage results in the lower amplitude and blue-shift of the C=O stretch peak,[8] it is very likely that the coverage of the lipid for the laser spot is drastically reduced, and thus the net surface charge in the probed region is significantly reduced in Ref. 6. To check the data reproducibility, we performed independent SFG measurements in the Ye group with the picosecond laser SFG system. The results are given with purple dots in Fig. S2. The data obtained from the Ye group also shows the characteristic frequency of ~1730 cm^{-1}. We used the rotational stage of the sample holder, avoiding the heat accumulation due to the laser radiation and thus allowing us to measure the SFG signal at the lipid interfaces without distortion of the lipid monolayer while the Tahara group did not rotate the samples during the measurements. Apparently, the different SFG response of the C=O stretch mode in Ref. 6 manifests that the formations of the lipid monolayer are significantly different.

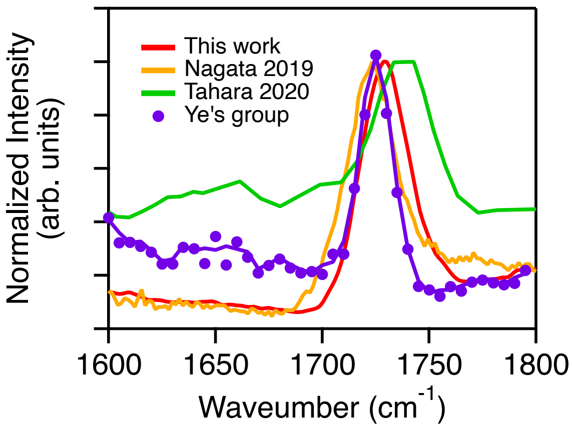

Figure S2. The $\left|\chi_{\mathrm{eff}}^{(2)}\right|^2$ spectra at the D_2O-DPTAP interfaces with salt concentrations of 1 M and 0.5 M (Tahara 2020). All the data sets are normalized to the intensity of the C=O stretch mode signal. The solid purple line is to guide the eye for Ye's group data. The data labeled as "Nagata 2019" and "Tahara 2020" are reproduced from Refs. [6] and [7], respectively.

Fitting Procedure for HD-SFG Spectra of Water Bending Mode

We performed the spectral fitting for the HD-SFG spectra (Im($\chi_{\mathrm{eff}}^{(2)}$) and Re($\chi_{\mathrm{eff}}^{(2)}$)) via

$$\chi_{\mathrm{eff}}^{(2)}(\omega) = A_{\mathrm{NR}} e^{i\phi_{\mathrm{NR}}} + \sum_{i=1,2} \chi_{\mathrm{C=O},i}^{(2)}(\omega) + \chi_{\mathrm{bend}}^{(2)}(\omega) + \chi_{\mathrm{bend}}^{(3)}(\omega)\,\Phi(\sigma, c)\frac{\kappa(c)}{\kappa(c) - i\Delta k_z} \tag{S1}$$

for the H_2O-lipid samples and via

$$\chi_{\mathrm{eff}}^{(2)}(\omega) = A_{\mathrm{NR}} e^{i\phi_{\mathrm{NR}}} + \sum_{i=1,2} \chi_{\mathrm{C=O},i}^{(2)}(\omega) \tag{S2}$$

for the D_2O- lipid samples. Here, A_{NR} (ϕ_{NR}), and $\chi^{(2)}_{\mathrm{C=O},i}(\omega)$ are the amplitude (phase) of the non-resonant contribution and the second-order susceptibility of the C=O stretch mode of carbonyl group of the lipid, respectively. $\chi^{(2)}_{\mathrm{bend}}(\omega)$ and $\chi^{(3)}_{\mathrm{bend}}(\omega)$ represent the second-order and third-order susceptibilities of the H-O-H bending mode. σ and c represent surface charge density and ion concentration. $\Phi(\sigma, c)$ and $\kappa(c)$ denote the surface potential and the inverse of the Debye length, respectively. Δk_z denotes the mismatch of the wave-vectors along z axis in the reflected SFG configuration.

In the Gouy-Chapman model, the surface potential $\Phi(\sigma, c)$ is given by[9]

$$\Phi_0(\sigma, c) = \frac{2k_BT}{e_c}\sinh^{-1}\left(\frac{\sigma}{\sqrt{8000k_BTN_A\varepsilon_0\varepsilon_r c}}\right) = 0.052 \cdot \sinh^{-1}\left(\frac{\sigma}{0.117\sqrt{c}}\right). \tag{S3}$$

The inverse of the Debye length $\kappa(c)$ is given by[9]

$$\kappa(c) = \sqrt{\frac{2000e_c^2N_Ac}{\varepsilon_0\varepsilon_r k_BT}} = \frac{\sqrt{c}}{0.304}, \tag{S4}$$

where k_B, T, e_c, N_A, ε_0, and ε_r represent the Boltzmann constant, temperature, the elementary charge, the Avogadro's number, the vacuum permittivity, and the relative permittivity of water, respectively. The surface charge density was assumed to be +0.35 C/m^2 and -0.30 C/m^2 for DPTAP and DPPG, respectively, based on the surface area per lipid. The mismatch of the wave-vectors, Δk_z, was calculated via;

$$\Delta k_z = k_{\mathrm{SFG},z} + k_{\mathrm{vis},z} + k_{\mathrm{IR},z}, \tag{S5}$$

and

$$k_{i,z} = \frac{2\pi}{\lambda}\sqrt{n_i^2 - sin^2\theta_i}\ \ (i\ =\ \mathrm{SFG, vis, or\ IR}),$$

(S6)

where $k_{i,z}$, λ, n_i, and θ_i indicate wave-vector at z-plane for the beam (*i*), wavelength, refractive index of water corresponding to the beam (*i*), and beam angle with respect to the surface normal, respectively. We obtained $\Delta k_z = 49$ (nm^{-1}).

For C=O stretch mode peaks ($\chi^{(2)}_{\mathrm{C=O,1}}(\omega)$ and $\chi^{(2)}_{\mathrm{C=O,2}}(\omega)$), we assumed a Lorentz shape

$$\chi^{(2)}_{\mathrm{C=O,i}}(\omega) = \frac{A_{\mathrm{C=O,i}}}{\omega - \omega_{\mathrm{C=O,i}} + i\Gamma_{\mathrm{C=O,i}}},$$

(S7)

where $A_{\mathrm{C=O},i}$, $\omega_{\mathrm{C=O},i}$, and $\Gamma_{\mathrm{C=O},i}$ denote the amplitude, characteristic frequency and a half width at half maxima of the *i*th C=O stretch mode, respectively (*i*=1,2).

For the water's bending mode contribution ($\chi^{(2)}_{\mathrm{bend}}(\omega)$ and $\chi^{(3)}_{\mathrm{bend}}(\omega)$), we assumed the Voigt profile;

$$\begin{aligned}\chi_{\mathrm{bend}}(\omega) &= \frac{A_{\mathrm{bend}}}{\omega - \omega_{\mathrm{bend}} + i\Gamma_{\mathrm{bend,hom}}} \otimes \frac{1}{\sqrt{2\pi}\Gamma_{\mathrm{bend,inh}}} \exp\left(-\frac{\omega^2}{2\Gamma^2_{\mathrm{bend,inh}}}\right) \\ &= \frac{1}{\sqrt{2\pi}\Gamma_{\mathrm{bend,inh}}} \int_0^\infty \frac{A_{\mathrm{bend}}}{\omega_{\mathrm{bend}} - \omega' + i\Gamma_{\mathrm{bend,hom}}} \exp\left(-\frac{(\omega-\omega')^2}{2\Gamma^2_{\mathrm{bend,inh}}}\right) d\omega',\end{aligned}$$

(S8)

where A_{bend}, ω_{bend}, and $\Gamma_{\mathrm{bend,hom}}$ denote the amplitude, characteristic frequency and homogeneous broadening, and $\Gamma_{\mathrm{bend,inh}}$ accounts for inhomogeneous broadening. To reduce the degrees of freedom on the fits, we assumed that $2\sqrt{2ln2}\Gamma_{\mathrm{bend,inh}}$ (full-width at half maxima of the gaussian distribution) and ω_{bend} for $\chi^{(3)}_{\mathrm{bend}}(\omega)$ were set to 35 cm^{-1} and 1650 cm^{-1}, respectively. We made constraints on $\Gamma_{\mathrm{bend,hom}}$ and $\Gamma_{\mathrm{bend,inh}}$ for the $\chi^{(2)}_{\mathrm{bend}}(\omega)$ contribution and A_{bend}, ω_{bend}, $\Gamma_{\mathrm{bend,hom}}$, and $\Gamma_{\mathrm{bend,inh}}$ for the $\chi^{(3)}_{\mathrm{bend}}(\omega)$ contribution to have the same values between the DPTAP and DPPG samples. The best fits were obtained with the $\Gamma_{\mathrm{bend,hom}}$ of 15 cm^{-1}.

Note that the fitting has been done with a Lorentzian lineshape in our previous study[5], whereas the $\mathrm{Im}\left(\chi_{\mathrm{eff}}^{(2)}(\omega)\right)$ spectra in the HD-SFG measurement shows that the bending mode contribution is not Lorentzian shape, rather close to the Gaussian shape. Thus, we decided to use the Voigt profile for fit the data.

To capture the H-O-H bending contribution, we performed a two-step fitting procedure for the $\mathrm{Im}\left(\chi_{\mathrm{eff}}^{(2)}(\omega)\right)$ spectra of the H_2O-lipid and D_2O-lipid interfaces via equations S1 and S2, respectively. With the shared information of $\omega_{\mathrm{C=O},i}$, and $\Gamma_{\mathrm{C=O},i}$, we performed global fits for the imaginary part SFG spectra with various salt concentrations in a similar manner to our previous work.[5] After extracting that the vibrationally-resonant responses from the imaginary part, we further performed fits for the $\mathrm{Re}\left(\chi_{\mathrm{eff}}^{(2)}(\omega)\right)$ spectra to describe the non-resonant contributions. Through the fits, we obtained $\chi_{\mathrm{bend}}^{(2)}(\omega) = \frac{1}{\sqrt{2\pi}15}\int_0^\infty \frac{0.021}{1672-\omega'+15\mathrm{i}} \exp\left(-\frac{(\omega-\omega')^2}{2\times15^2}\right) d\omega'$ for the water-DPTAP interface, $\chi_{\mathrm{bend}}^{(2)}(\omega) = \frac{1}{\sqrt{2\pi}15}\int_0^\infty \frac{-0.13}{1652-\omega'+15\mathrm{i}} \exp\left(-\frac{(\omega-\omega')^2}{2\times15^2}\right) d\omega'$ for the water-DPPG interface, and $\chi_{\mathrm{bend}}^{(3)}(\omega) = \frac{1}{\sqrt{2\pi}15}\int_0^\infty \frac{-0.54}{1650-\omega'+15\mathrm{i}} \exp\left(-\frac{(\omega-\omega')^2}{2\times15^2}\right) d\omega'$. The fitting curves are plotted in Figures S1 and S2, and the results are summarized in Tables S1 and S2.

The obtained sign of the $\mathrm{Im}\left(\chi_{\mathrm{bend}}^{(2)}(\omega)\right)$ and $\mathrm{Im}\left(\chi_{\mathrm{bend}}^{(3)}(\omega)\,\Phi(c)\right)$ spectra are consistent with our previous study.[5] However, critical comparison of the current data set obtained from the HD-SFG with the previous data set obtained from the homodyne-detected SFG indicates that some fit parameters are not accurately obtained because of the uncertainty of the fit. First, a broad peak feature at ~1800 cm^{-1} assumed based on Ref. 8 is not present in the current HD-SFG data. This misleadingly assumed 1800 cm^{-1} peak affects the fit of the non-resonant background. Second, the characteristic frequency of the obtained $\chi_{\mathrm{bend}}^{(2)}(\omega)$ peak at the water-DPTAP interface was ~1645 cm^{-1} in our previous study, while the frequency obtained in this study was ~1670 cm^{-1}. These illustrate that, for rigorous discussion of the bending mode frequency, the HD-SFG technique is highly required.

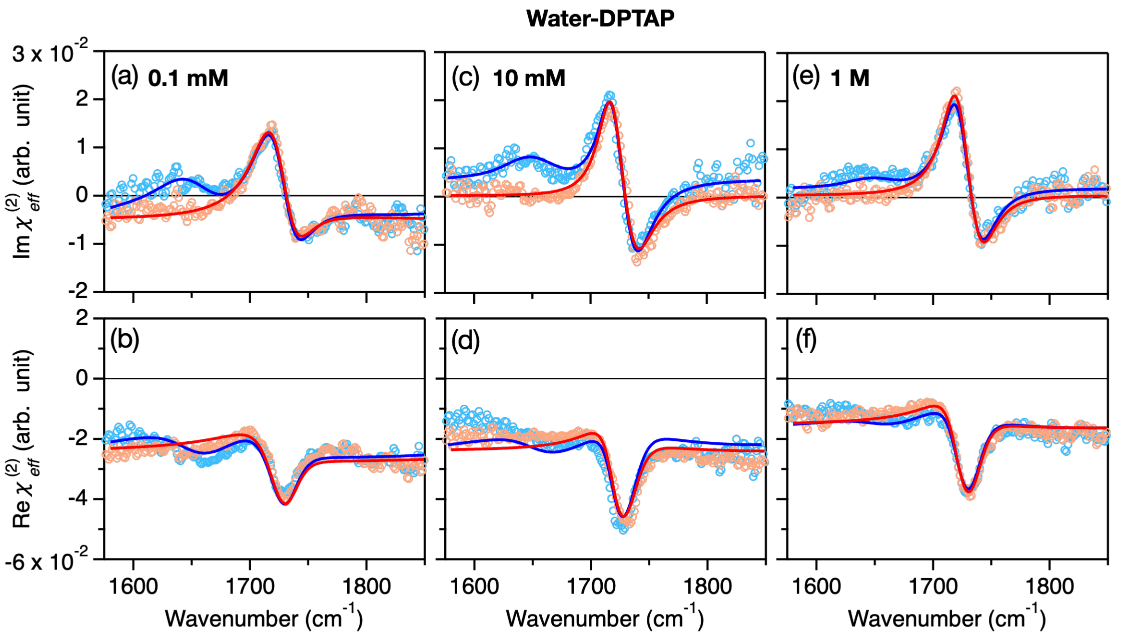

Figure S3. (a-f) HD-SFG spectra at the H_2O-DPTAP interface (blue) and at the D_2O-DPTAP interface (red) with various NaCl concentrations. The solids lines represent the fits.

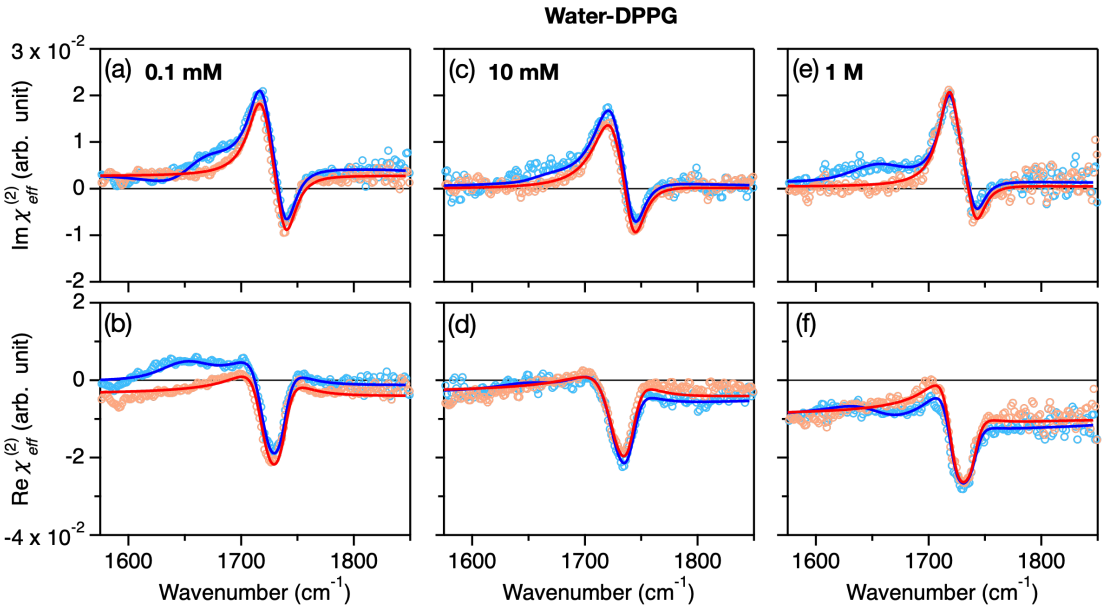

Figure S4. (a-f) HD-SFG spectra at the H_2O-DPPG interface (blue) and at the D_2O-DPPG interface (red) with various NaCl concentrations. The solids lines represent the fits.

Table S1. The fitting parameters of the complex SFG spectra at water-DPTAP interfaces. The error was obtained from the fits.

DPTAP	H_2O	D_2O	H_2O	D_2O	H_2O	D_2O
NaCl	1 M		10 mM		0.1 mM	
A_{NR}	0.016 ± 0.001	0.016 ± 0.001	0.023 ± 0.001	0.024 ± 0.001	0.024 ± 0.001	0.026 ± 0.001
ϕ_{NR} (rad)	3.03 ± 0.01	3.12 ± 0.01	2.98 ± 0.03	3.13 ± 0.04	3.30 ± 0.04	3.34 ± 0.03
$A_{C=O,1}$	-0.45 ± 0.08	-0.51 ± 0.08	-0.42 ± 0.05	-0.45 ± 0.05	-0.71 ± 0.15	-0.72 ± 0.14
$\omega_{C=O,1}$ (cm^{-1})	1720.7 ± 1.0		1718.5 ± 0.7		1721.3 ± 2.5	
$\Gamma_{C=O,1}$ (cm^{-1})	16.4 ± 1.0		14.7 ± 0.9		23.2 ± 1.5	
$A_{C=O,2}$	0.38 ± 0.08	0.39 ± 0.08	0.48 ± 0.05	0.41 ± 0.05	0.48 ± 0.14	0.45 ± 0.13
$\omega_{C=O,2}$ (cm^{-1})	1738.5 ± 1.6		1736.4 ± 1.2		1736.0 ± 2.2	
$\Gamma_{C=O,2}$ (cm^{-1})	17.8 ± 1.3		19.3 ± 1.1		20.3 ± 2.2	
A_{bend} for $\chi^{(2)}_{bend}$	0.021 ± 0.007	NA	0.021 ± 0.007	NA	0.021 ± 0.007	NA
ω_{bend} for $\chi^{(2)}_{bend}$	1672.0 ± 0.9	NA	1672.0 ± 0.9	NA	1672.0 ± 0.9	NA
A_{bend} for $\chi^{(3)}_{bend}$	-0.541 ± 0.031	NA	-0.541 ± 0.031	NA	-0.541 ± 0.031	NA

Table S2. The fitting parameters of the complex SFG spectra at water-DPPG interfaces. The error was obtained from the fits.

DPPG	H_2O	D_2O	H_2O	D_2O	H_2O	D_2O
NaCl	1 M		10 mM		0.1 mM	
A_{NR}	0.011 ± 0.001	0.009 ± 0.001	0.004 ± 0.001	0.003 ± 0.001	0.004 ± 0.001	0.005 ± 0.001
ϕ_{NR} (rad)	3.02 ± 0.01	3.10 ± 0.01	3.03 ± 0.03	3.16 ± 0.04	1.81 ± 0.04	2.52 ± 0.03
$A_{C=O,1}$	-0.24 ± 0.01	-0.27 ± 0.01	-0.40 ± 0.05	-0.36 ± 0.05	-0.28 ± 0.02	-0.28 ± 0.02
$\omega_{C=O,1}$ (cm^{-1})	1718.9 ± 0.4		1723.4 ± 1.4		1717.7 ± 0.8	
$\Gamma_{C=O,1}$ (cm^{-1})	12.0 ± 0.6		19.3 ± 1.1		14.2 ± 0.8	
$A_{C=O,2}$	0.11 ± 0.01	0.12 ± 0.01	0.22 ± 0.04	0.23 ± 0.04	0.20 ± 0.02	0.21 ± 0.02
$\omega_{C=O,2}$ (cm^{-1})	1741.6 ± 0.7		1742.9 ± 0.7		1738.9 ± 0.6	
$\Gamma_{C=O,2}$ (cm^{-1})	10.7 ± 1.0		12.7 ± 1.1		12.0 ± 0.8	
A_{bend} for $\chi^{(2)}_{bend}$	-0.130 ± 0.007	NA	-0.130 ± 0.007	NA	-0.130 ± 0.007	NA
ω_{bend} for $\chi^{(2)}_{bend}$	1652.4 ± 1.1	NA	1652.4 ± 1.1	NA	1652.4 ± 1.1	NA
A_{bend} for $\chi^{(3)}_{bend}$	-0.541 ± 0.031	NA	-0.541 ± 0.031	NA	-0.541 ± 0.031	NA

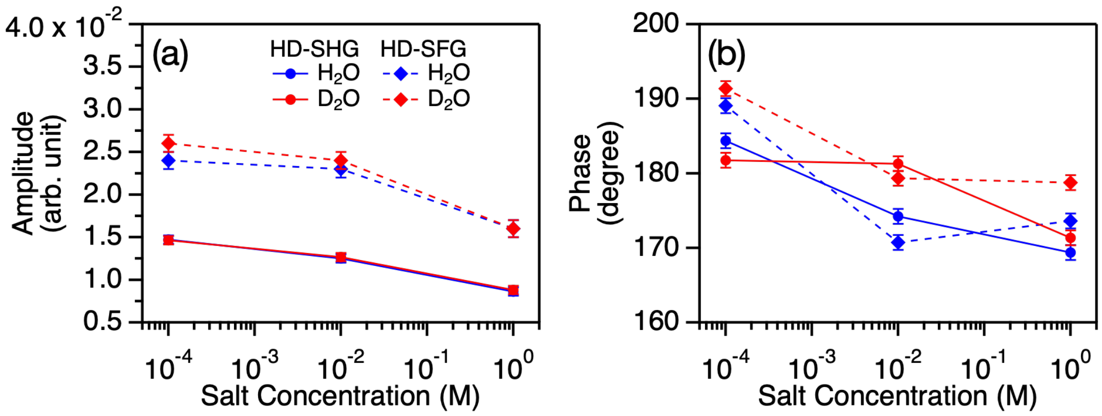

Figure S5. (a, b) Amplitude (a) and phase (b) of the non-resonant contribution obtained by HD-SHG as well as the fit to the HD-SFG spectra for the water-DPTAP interface (Table S1). The error bars indicate the 95% confidence intervals. The 10 mM data are displayed in Figure S3 in the Supporting Information.

Phase Accuracy of the collinear HD-SFG measurement

To evaluate the phase accuracy of our HD-SFG measurement, we measured D_2O-air interface sample without lipid monolayer. Figure S6 shows the real part and the imaginary part spectra of the measured $\chi^{(2)}_{\mathrm{eff}}(\omega)$. Because there is no specific vibrational mode at D_2O-air interface, the spectral region of interest is dominated only by the non-resonant contribution, displaying zero contribution in the imaginary part. As such, we conclude that no phase calibration is needed.

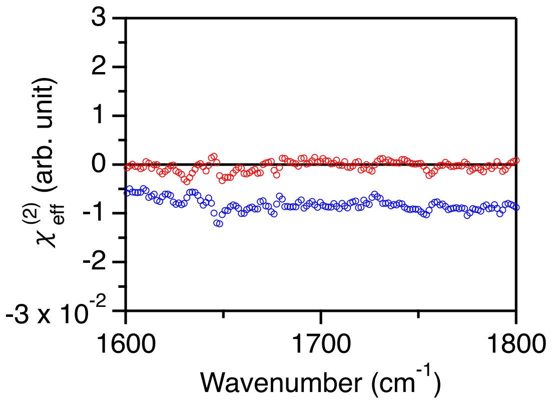

Figure S6. HD-SFG spectra at the D_2O-air interface. The blue and red data points indicate the real and imaginary part, respectively.

REFERENCES

(1) Sung, W.; Seok, S.; Kim, D.; Tian, C. S.; Shen, Y. R. Sum-Frequency Spectroscopic Study of Langmuir Monolayers of Lipids Having Oppositely Charged Headgroups. *Langmuir* **2010**, *26*, 18266–18272.

(2) Liu, W.; Wang, Z.; Fu, L.; Leblanc, R. M.; Yan, E. C. Y. Lipid Compositions Modulate Fluidity and Stability of Bilayers: Characterization by Surface Pressure and Sum Frequency Generation Spectroscopy. *Langmuir* **2013**, *29*, 15022–15031.

(3) Backus, E. H. G.; Bonn, D.; Cantin, S.; Roke, S.; Bonn, M. Laser-Heating-Induced Displacement of Surfactants on the Water Surface. *J. Phys. Chem. B* **2012**, *116*, 2703–2712.

(4) Nihonyanagi, S.; Mondal, J. A.; Yamaguchi, S.; Tahara, T. Structure and Dynamics of Interfacial Water Studied by Heterodyne-Detected Vibrational Sum-Frequency Generation. *Annu. Rev. Phys. Chem.* **2013**, *64*, 579–603.

(5) Seki, T.; Sun, S.; Zhong, K.; Yu, C.; Machel, K.; Dreier, L. B.; Backus, E. H. G.; Bonn, M.; Nagata, Y. Unveiling Heterogeneity of Interfacial Water through the Water Bending Mode. *J. Phys. Chem. Lett.* **2019**, *10*, 6936–6941.

(6) Ahmed, M.; Nihonyanagi, S.; Kundu, A.; Yamaguchi, S.; Tahara, T. Resolving the Controversy over Dipole versus Quadrupole Mechanism of Bend Vibration of Water in Vibrational Sum Frequency Generation Spectra. *J. Phys. Chem. Lett.* **2020**, 9123–9130.

(7) Seki, T.; Sun, S.; Zhong, K.; Yu, C. C.; MacHel, K.; Dreier, L. B.; Backus, E. H. G.; Bonn, M.; Nagata, Y. SI- Unveiling Heterogeneity of Interfacial Water through the Water Bending Mode. *J. Phys. Chem. Lett.* **2019**.

(8) Dreier, L. B.; Bonn, M.; Backus, E. H. G. Hydration and Orientation of Carbonyl Groups in Oppositely Charged Lipid Monolayers on Water. *J. Phys. Chem. B* **2019**, *123*, 1085–1089.

(9) Bard, A. J.; Faulkner, L. R. *Electrochemical Methods: Fundamentals and Applications*; wiley New York, 2001; Vol. 2.
